# One Yeast, Sixteen Synthetic Chromosomes, Infinite Possibilities

**DOI:** 10.1002/yea.70026

**Published:** 2026-05-19

**Authors:** Edward Archer, Roy S. K. Walker, Paige E. Erpf, Ian T. Paulsen, Isak S. Pretorius

**Affiliations:** ^1^ ARC Centre of Excellence in Synthetic Biology Macquarie University Sydney New South Wales Australia; ^2^ The Australian Genome Foundry Sydney New South Wales Australia; ^3^ The Chancellery Macquarie University Sydney New South Wales Australia

**Keywords:** minimal genome, pan‐genome, *Saccharomyces cerevisiae* synthetic genome Sc2.0, supernumerary neochromosomes, synthetic biology

## Abstract

The evolution of the yeast, *Saccharomyces cerevisiae*, from a genetically tractable model organism to a chassis for genome‐scale engineering represents one of the most influential trajectories in eukaryotic biology. The Synthetic Yeast Genome Project (Sc2.0) embodies the current height of this trajectory, having now delivered functional synthetic versions of all 16 native yeast chromosomes and bringing the construction of the first fully synthetic eukaryotic cell within reach. Beyond its technical achievements, Sc2.0 has reshaped how eukaryotic genomes are understood and explored through iterative design‐build‐test‐learn (DBTL) cycles, and reframed the yeast genome as a dynamic, highly modifiable system rather than a static biological blueprint. Moreover, the progress on genome engineering pipelines and synthetic biology has laid the foundations for the de novo development of modular synthetic chromosomes (neochromosomes) that operate orthogonally to the native genome. These synthetic platforms provide dedicated, large‐scale genomic landing pads for refactoring genetic networks, reallocating redundancy, and introducing large, multiplexed gene assemblies, thereby extending yeast engineering toward programmable and hyper‐versatile biological systems. To commemorate the 40th anniversary of the journal *Yeast*, this minireview celebrates the exceptional power of yeast genetics, outlining key conceptual and technological advances emerging from the Sc2.0 endeavour and beyond. Finally, we examine the cross‐cutting engineering insights and the future potential of neochromosomes for the next generation of synthetic yeasts.

## Summary

- Sc2.0 delivered 16 functional synthetic yeast chromosomes.
- Sc2.0 brings a first fully synthetic eukaryotic cell within reach.
- Sc2.0 reframed the yeast genome as a dynamic, programmable system.
- Neochromosomes enable modular, large‐scale genome design.

## 1 Introduction

From its earliest domestication in baking, brewing, and winemaking, yeast has served as one of humanity's oldest biotechnological tools. Its transformation from a fermentation workhorse into a central experimental model, however, arose from a series of conceptual breakthroughs during the late 19th century. Through Pasteur's demonstration that fermentation is a biological process, Hansen's development of pure‐culture isolation methodologies, and Buchner's discovery that fermentation could occur in cell‐free extracts, yeasts were progressively being established as experimentally tractable biological systems at a time before the underlying genetic and biochemical mechanisms were well understood (Nielsen 2025). As such, the extended cultural and evolutionary entanglement of yeasts laid the foundation for their ultimate transition from a mere fermentation workhorse to an organism of exceptional scientific and industrial value.

As experimental biology expanded in the early 20th century, *Saccharomyces cerevisiae* emerged from among more than 1,500 described species as the most intensively studied and widely harnessed representative, which earned it the reputation as the ‘Swiss Army knife’ (or omni‐tool) of the microbial world (Pretorius 2017). A defining feature of *S. cerevisiae* as an experimental model is its relative structural and genetic simplicity, which enables seamless integration of flexible technologies whilst also supporting highly reproducible experimental research. The cell could be viewed as a genetic system, a metabolic network, or a biochemical reactor without losing coherence as a model. Consequently, *S. cerevisiae* did not become a powerful model because it was engineered to be so; rather, it was extensively engineered because it had already proven itself over decades of experimentation. This success arose from its rapid growth, modest cultivation requirements, its ‘generally regarded as safe’ (GRAS) status, and the domestication of laboratory strains that enabled methodological standardisation across the field (Nielsen 2025).

This review traces how these intrinsic attributes positioned *S. cerevisiae* to progress beyond conventional genome manipulation and genetic engineering toward large‐scale synthetic genome writing and synthetic yeast cells. We examine how advances in DNA assembly and genome writing have transformed *S. cerevisiae* into a living genome foundry, capable of assembling kilobase‐ to megabase‐scale DNA constructs and generating modular, de novo designed synthetic chromosomes (neochromosomes) with broad and scalable biotechnological potential.

## 2 Essential Features of a Living Genome Foundry

Throughout the latter half of the 20th century, advances in yeast genetics converged on a defining realisation, namely the exceptionally efficient homologous recombination (HR) machinery of *S. cerevisiae*. In most eukaryotes and a wide range of yeast species, non‐homologous end joining (NHEJ) serves as the predominant DNA repair mechanism (Dudášová et al. 2004; Sonoda et al. 2001). While vital for natural genomic integrity, NHEJ constrains precise genetic engineering by facilitating the ligation of DNA ends with minimal sequence homology. This lack of specificity often results in the random integration of exogenous DNA at unintended loci or the introduction of deleterious small insertions and deletions (indels) that can disrupt the structural and functional integrity of synthetic genes and regulatory elements.

Many industrially relevant non‐conventional yeasts, such as the methylotrophic yeast *Komagataella phaffii* and the oleaginous yeast *Yarrowia lipolytica*, remain natively NHEJ‐dominant (Ji et al. 2020; Zhou et al. 2025). Overcoming this repair constraint to improve engineering precision in these species typically requires the deletion of key NHEJ regulators, such as the developing ΔKU70 or ΔKU80 mutant strains, to artificially shift the repair bias toward homologous recombination. The necessity of such foundational genetic modifications in other species further underscores the unique value of *S. cerevisiae* as a specialized and tractable host for large‐scale synthetic genomics. In contrast, NHEJ is considered a secondary repair mechanism, with the exceptionally efficient HR machinery as its primary repair modality (Chovanec and Wilson 2007; Dudášová et al. 2004; Sonoda et al. 2001). This has been showcased by the frequency of targeted integration in this yeast species, where HR‐mediated DNA repair is orders of magnitude higher than in mammalian cells, and can reach successful recombination of heterologous gene cassettes into its genome with fewer than 100 base pairs (Sonoda et al. 2001).

The inherent capability of *S. cerevisiae* to facilitate the seamless joining of multiple overlapping DNA fragments using HR has established this eukaryotic model as a premier ‘factory’ for complex genome engineering. Moreover, unlike in vitro ligation‐based recombination methods, this assembly is performed within a living eukaryotic cell, where recombination is co‐facilitated by DNA replication and repair pathways. This property underpins two conceptually distinct yet technically related genome writing capabilities in *S. cerevisiae*: (i) serving as a highly efficient in vivo DNA assembly platform for large exogenous constructs, and (ii) enabling precise and stable modification of its own genome.

### 2.1 In Vivo Assembly of Large DNA Fragments

The characterisation of essential *cis*‐acting chromosomal elements, including centromeres, autonomous replicating sequences (ARS), and telomeres, provided the molecular elements machinery required to develop yeast shuttle vectors and yeast artificial chromosomes (YAC). These essential genomic elements and vectors allow for the stable maintenance of large exogenous DNA fragments in *S. cerevisiae* (Burke et al. 1987; Ma et al. 1987; Murray & Szostak. 1983; Myers et al. 1986). This capacity positions *S. cerevisiae* either as an intermediate chassis for assembling large DNA constructs intended for transfer into other organisms or as a host for maintaining a substantial amount of additional DNA independently from its native genome.

A key advantage of this platform lies in its potential for modularity. DNA fragments derived from diverse heterologous origins can be assembled or recombined within a single eukaryotic host. This capability has enabled the development of various methodologies for reconstructing complete viral and bacterial genomes, developing human artificial chromosomes (HAC), and capturing genomic loci from higher eukaryotes, such as algae, plants, and mammals (Table 1). With several methodologies that were developed in tandem to isolate and deliver such in vivo‐assembled DNA components to recipient cells, *S. cerevisiae* serves as a universal assembly environment for chromosome‐scale constructs.

**Table 1:** Examples of research utilising the strong homologous recombination (HR) machinery of *Saccharomyces cerevisiae* to clone and assemble large segments of DNA for downstream applications.

DNA origin	Destination cell type	Assembly method	Product size (kb)	Ref
**Virus**				
Dengue Virus 2	Mammalian (LLC‐MK_2_)	4x viral cDNA fragments (~2.5–3 kb) assembly onto a YAC.	~10	Polo et al. (1997)
SARS‐Cov‐2	Bacteria (*Escherichia coli*)	6x cDNA fragments (~5 kb) assembly into a YAC.	~30	Xiong et al. (2024)
Mouse hepatitis virus; SARS‐Cov‐2	Mammalian (Vero B4, Vero E6, L929)	~10 cDNA fragments from viral RNA (~0.5–3 kb) and cloned in yeast using TAR cloning.	~30	Thi Nhu Thao et al. (2020)
*Haemophilus influenzae*	Human (U2OS, HT1080)	~10–12x genomic fragments (~150–200 kb) plus ~5–10x human centromeric modules (~10–50 kb) assembled into a YAC.	~2000	Birchak et al. (2026)
HSV‐1; EBOV (VP35 protein sequence)	Mammalian (HEK293, Vero, HeLa)	Bacterial filler DNA (~100 kb–1 Mb) and HSV‐1 genome (152 kb) assembled onto yeast centromeric plasmids (YCp) using TAR cloning.	~1100	Brown et al. (2017)
**Bacteria**				
*Mycoplasma genitalium*	Bacteria (*M. genitalium*)	In vitro recombination of 101x fragments (5–7 kb) into 4x chunks (~144 kb), cloned into a BAC. 4x chunks assembled in yeast using TAR cloning.	~580	Gibson et al. (2008)
*M. mycoides*	Bacteria (*M. capricolum*)	1078x synthetic bacterial DNA fragments (~1 kb) recombined stepwise to form 11x intermediate segments (~100 kb), followed by full genome assembly.	~1,080	Gibson et al. (2010)
*M. mycoides*	Bacteria (*M. mycoides)*	5x DNA fragments (1.4 kb) assembled into 7 kb chunks, followed by 15x chunks assembled into ~100 kb DNA cassettes.	~530	Hutchison et al. (2016)
*E. coli*	Bacteria (*E. coli*)	7–14x synthetic DNA chunks (each 6–13 kb) assembled into a BAC.	91–136	Fredens et al. (2019)
*Synechococcus elongatus*	Bacteria (*E. coli*)	30x genomic DNA fragments (87–134 kb) cloned in yeast using TAR. Assembly of 5x genomic DNA fragments (~100 kb) onto a YAC containing 3x ARS sequences.	~454	Noskov et al. (2012)
**Fungal**				
*Saccharomyces cerevisiae*	*N/A*	171x fragments' *(*~5 kb) assembled into 18x chunks (30–60 kb), followed by successive chromosomal replacement and pooled PCRTag mapping for debugging.	~700	Wu et al. (2017)
*S. cerevisiae*	*N/A*	Three rounds of stepwise YLC‐assembly of 23, 48, and 95 kb, respectively.	95	He et al. (2023)
*S. cerevisiae*	*N/A*	Assembly of tRNA arrays (~10 kb) across the yeast genome onto a yeast centromeric vector.	~190	Schindler et al. (2023)
*S. cerevisiae*	*N/A*	17x pan‐genome sequences (75 ORFs; 1.1–60 kb) assembled onto a yeast centromeric vector.	220	Kutyna et al. (2022)
*S. cerevisiae*	*N/A*	23‐44x bacterial filler DNA segments (~2.5 kb), essential yeast chromosomal elements and 13X yeast glycolytic/fermentative gene cassettes (35 kb) assembled as a synthetic chromosome.	85–135	Postma et al. (2021)
**Algae**				
*Phaeodactylum tricornutum*	*N/A*	5x fragments assembled into ~100 kb genomic chunks using TAR cloning and HR–mediated assembly.	~440–500	Karas et al. (2013)
*P. tricornutum*	*N/A*	8–20x overlapping chloroplast DNA fragments (~2.5–17.6 kb) with a BAC‐based vector.	~120	Walker et al. (2024)
**Plant**				
*Zea mays*	*N/A*	Size‐selected ~100–200 kb genomic fragments ligated into a modified YAC vector, followed by stable maintenance of a~139 kb chloroplast genome in yeast.	~139	Gupta and Hoo (1991)
*Physcomitrium patens*	*Plant (P. patens)*	10x fragments (~3 kb) assembled to create 3x mid‐sized DNA chunks (~30 kb) to yield a final mega‐chunk (~100 kb) onto a YCp/BAC vector, followed by PEG‐mediated transformation to the recipient host plant cell.	~70	Chen et al. (2024)
**Mammalian**				
*Homo sapiens*	Mouse (embryonic stem cells)	38x amplicons (~3 kb) divided into three for iterative extrachromosomal SwAP‐In (eSwAP‐In) assembly in yeast.	~101	Mitchell et al. (2021)
*H. sapiens* (β‐globin locus)	Mouse (MEL cell line)	Size‐selected high molecular weight genomic fragments cloned into a YAC vector for stable maintenance and transfer to mammalian cells.	248	Peterson et al. (1993)
*Rat (synthetic HoxA locus)*	Mouse (embryonic stem cells)	28x PCR amplicons (~5 kb) assembled onto a yeast assembly vector (pLM453).	130–170	Pinglay et al. (2022)
*H. sapiens* (IGH locus)	*N/A*	YLC‐assembly of 424, 426, and 407 kb fragments of the IGH locus, followed by 833 kb assembly, and final 1.26 Mb assembly.	~1,260	He et al. (2023)
*H. sapiens*	Mouse early embryos	233x synthetic fragments of the human *hAZFα* locus (~5.5 kb) assembled stepwise into ~40–70 kb chunks and ~270–330 kb megachunks, and finally 1.14 Mb constructs, combined with yeast mating & CRISPR/Cas9‐assisted recombination.	1,140	Liu et al. (2025)
Mouse & *H. sapiens*	*N/A*	Human gDNA isolated from lymphoblastoid cell line & high molecular weight gDNA from mouse spleen tissue, and variable‐sized fragments assembled onto YACs.	~200–2000	Larin et al. (1991)
*H. sapiens*	Human (HT1080 & U2OS cell lines)	Filler DNA from *M. mycoides* (~550 kb) assembled on a YAC alongside human chromosome segment 4q21 (182 kb) using TAR.	~750	Birchak et al. (2025); Gambogi et al. (2024)
*H. sapiens*	*S. cerevisiae* & *M. musculus* genome integration	Commercial DNA synthesis of 28x–36x fragments (~1.3–4 kb) of a reversed HPRT1 locus (HPRT1R) and assembled onto a yeast assembly vector (YAV).	~100	Camellato et al. (2024)

### 2.2 Precision Genome Engineering

Early demonstrations of *S. cerevisiae* genome modification established that linear DNA fragments bearing homologous flanking regions as little as 30–50 bp could replace existing DNA or insert heterologous gene cassettes at chromosomal loci with high precision and fidelity (Hinnen et al. 1978; Ma et al. 1987; Orr‐Weaver et al. 1981; Szostak et al. 1983).

Subsequent refinements in engineering toolkits that further utilise this exceptional HR machinery in *S. cerevisiae* include counter‐selection systems, inducible expression systems, and marker recycling strategies, which expanded the scale and precision of genome manipulation (Boeke et al. 1984; Cameron et al. 1979; Colleaux et al. 1988; Sakai et al. 1990; Warmington et al. 1985). As a result, defined genetic modifications could be introduced that enable iterative cycles of genetic manipulation, rendering the yeast genome a dynamic engineering substrate. This property enabled the introduction of multi‐gene cassettes, pathway refactoring, and chromosomal rearrangements long before genome editing became routine in other eukaryotic models.

Importantly, yeasts endogenous recombination machinery is efficient enough to support large‐scale modifications without the need to cleave DNA with exogenous nucleases or gene editing tools. This property has enabled the introduction of multi‐gene cassettes, pathway refactoring, and chromosomal rearrangements long before genome editing became routine in other eukaryotes. It was only more recently that the recombination engine was harnessed as the essential repair machinery for emerging CRISPR‐based editing and other large‐scale genome writing platforms (Chen et al. 2025; Giersch and Finnigan 2017). By the late 20th century, *S. cerevisiae* was already uniquely positioned at the intersection of tailored genome manipulation and genome synthesis. However, the transition to large‐scale genome editing and rewriting was constrained by the need for a comprehensive understanding of chromosome organisation and gene content. In the absence of a complete genome sequence, genetic manipulation enabled local precision but did not allow coordinated, system‐level modifications within a fully defined genomic framework.

## 3 From Mapping to Refactoring Entire Yeast Genomes

The transition from yeast as a genetically tractable organism to a genome‐scale engineering platform was facilitated by a technological shift from partial genomic maps to complete chromosomal sequence resolution. The defining milestone was reached in 1992 with the first fully sequenced eukaryotic chromosome, that of *S. cerevisiae* chromosome III (Oliver et al.^1992^). For the first time, a chromosome landscape could be examined in its entirety, setting the conceptual and technical foundation for mapping whole eukaryotic genomes. Then, the Yeast Genome Sequencing Project was concluded in 1996, marking a defining moment in eukaryotic biology by delivering the first fully sequenced eukaryotic genome and providing a complete genetic map of the ~12 Mb haploid S. cerevisiae S288c lineage, including strains FY1679 and AB972 (Dujon and Cooper^2026^; Engel et al. 2014; Goffeau et al. 1996). For the first time, all protein‐coding genes, regulatory elements, and chromosomal features could be interrogated in a unified framework. The availability of a complete genome fundamentally altered experimental approaches, where genetic modifications could be contextualised within a fully defined genomic landscape.

Our understanding of the yeast genome expanded considerably as research attempted to understand more of the mechanisms related to gene redundancy (Dujon 1996; Goffeau et al. 1996). Consequently, a large scale of resources, including gene deletion strain collections (Giaever and Nislow 2014) and systematic genetic interaction mapping (Costanzo et al. 2016) enabled further characterisation of gene function across diverse conditions (Bosch‐Guiteras and van Leeuwen 2022; Svensson et al. 2011). Subsequent population‐scale genomics (pan‐genomics) further reinforced this view, whereby comparative analyses across diverse *S. cerevisiae* lineages uncovered extensive genetic and phenotypic diversity, reframing the yeast genome as a continually evolving entity shaped by duplication, adaptation, and contingency (Loegler et al. 2024; Peter et al. 2018; Wang et al. 2025a; Wang et al. 2024).

These insights had profound implications for the field of synthetic biology. If natural evolution tolerates and exploits large‐scale genomic restructuring, then deliberate and rational restructuring of the native genome may likewise be biologically feasible. Moreover, if substantial portions of the genome are dispensable under defined culturing conditions, then the genome itself becomes a substrate for significant redesign. Redundant sequences can be removed, non‐essential regions reorganised or repurposed as integration sites, and chromosomal structure streamlined to enhance stability. As a result, fundamental questions about eukaryotic genome organisation can be approached experimentally in conjunction with theory. By the onset of the 21st century, the intellectual and technical prerequisites to rewrite entire eukaryotic genomes were in place. These included a complete genome sequence with relatively comprehensive functional annotations, scalable DNA assembly methodologies, and a host organism (*S. cerevisiae*) with an exceptional track record of recombination.

## 4 (Re)Writing a Eukaryotic Genome: The SC2.0 Project

Together with full genomic maps and advanced genetic engineering toolkits, a conceptual shift towards constructing complete synthetic yeast chromosomes. This was deemed the logical progression following the successful assembly of synthetic bacterial genomes, in which *S. cerevisiae* also served a complementary role as a genome foundry (Gibson et al. 2008; Gibson et al. 2010). The first major milestone was reported in 2011 with the construction of fully synthetic and semi‐synthetic chromosome arms (*SynIXR* and *semi‐SynVIL*), demonstrating that large genomic regions could be rewritten while preserving cellular viability (Dymond et al. 2011).

**Figure 1:**
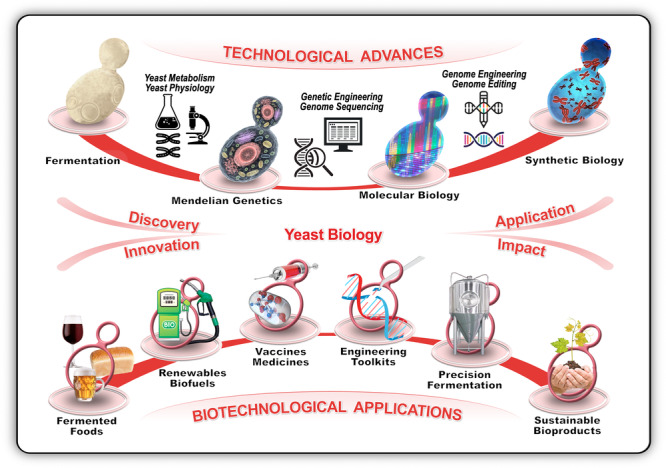
The evolution of the model yeast *Saccharomyces cerevisiae* over the decades reflects the progression of modern biotechnology. Early technological advances in taxonomy and fermentation treated yeast largely as a ‘fermentation black box’ to produce fermented beverages and foodstuffs. Advances in molecular biology, genetics, and sequencing throughout the 20th century enabled detailed characterisation of yeast genetic makeup and fundamental eukaryotic processes, establishing yeast as a premier model system and platform for heterologous gene expression and metabolic pathway engineering to generate value‐added compounds. The onset of the 21st century ushered in the synthetic biology era, defined by advanced design‐build‐test‐learn (DBTL) cycles for large‐scale genome writing, further facilitated by computational design and laboratory automation. This transformed yeast into a programmable chassis for diverse and sustainable biotechnological applications and for deeper exploration of fundamental principles of eukaryotic biology.

Following this achievement, an international consortium was launched to construct the worlds first fully synthetic eukaryotic genome. The *Synthetic Yeast Genome Project* (*Sc2.0;* www.syntheticyeast.org) initiative was made possible by a globally distributed network of research groups, each contributing specialised expertise and a shared vision of constructing the sixteen synthetic chromosomes of the *S. cerevisiae* genome (Annaluru et al. 2014; Dymond et al. 2011; Pretorius & Boeke. 2018; Richardson et al. 2017). Ultimately, the Sc2.0 project distinguished itself as a testbed for the next generation in synthetic biology discoveries (Figure 1), where advances in synthetic genome design and their resulting biotechnological potential hold substantial promise for broad societal impact (Pretorius & Boeke. 2018).

### 4.1 Cross‐Cutting Insights Uncovered by Sixteen Synthetic Yeast Chromosomes

The Sc2.0 project provided a systems‐level framework for eukaryotic genome rewriting. Moreover, the highly efficient HR machinery in *S. cerevisiae* proved to be a central component to this effort, combined with a hierarchical SwAP‐In strategy for iterative replacement of native loci with tailored synthetic counterparts (Richardson et al. 2017). Computer‐aided design principles were also developed, namely the BioStudio platform, which facilitated the ambitious genome‐wide redesign within the project consortium. Moreover, a suite of engineering principles was used to facilitate the removal of repetitive DNA, streamlining of intergenic regions, reassignment of stop codons, the strategic insertion of LoxPsym sites to enable synthetic chromosome rearrangement and modification by LoxP‐mediated evolution (SCRaMbLE), and embedding PCR tags to facilitate rapid genotypic tracking by distinguishing between synthetic and native sequences (Dymond et al. 2011).

The SCRaMbLE system, induced by Cre recombinase, targets loxPsym sites that are distributed throughout a synthetic chromosome or vector containing large sets of synthetic DNA parts. As a result, a massive variety of stochastic events unfold at these recombination sites, including indels, inversions, duplications, and translocations. This effectively allows for a so‐called ‘evolution on demand,’ generating a massive library of genotypes in a single culture, from which the generated populations can then be screened for a desired phenotype outcome, such as increased metabolic flux, stress tolerance, or alternative substrate utilisation (Blount et al. 2018).

In addition, researchers can use the Dre‐rox system as an orthogonal alternative or complement to the Cre‐loxPsym system (Liu et al. 2018; Schindler et al. 2023). Because these two systems do not cross‐react, and their induction can be controlled by two separate binding domains, such as a progesterone‐binding domain (PBD) and β‐estradiol‐binding domain (EBD), they enable a form of multi‐recombinase SCRaMbLE, resulting in independent stochastic events to be induced on synthetic DNA constructs (Liu et al. 2018).

In addition, with highly transcribed tRNA gene loci across the genome that are considered as hotspots of recombination and genomic instability, Sc2.0 design principles dictated their relocation onto a dedicated, de novo‐designed neochromosome (Schindler et al. 2023). This demonstrated that dispersed, yet essential molecular elements can be consolidated onto an orthogonal chromosomal platform.

Genome stability became both a central design consideration and an engineering constraint. Synthetic chromosome assembly throughout the Sc2.0 research consortia clarified many of the limitations that can be experienced in large‐scale eukaryotic genome engineering. These collective findings are reviewed in greater detail by Erpf et al. (2025), which summarises the overall successes and challenges experienced during the Sc2.0 project. These identified limitations further provided novel insights into gene function and genome structure. In addition to defects arising from synthetic genome design and construction, recurrent fitness phenotypes observed across independently engineered chromosomes (i.e., across different research groups) suggest the presence of intrinsic biological constraints embedded within the native genome itself. The emergent phenotypic and strain fitness bugs from the various engineered strains across the Sc2.0 research consortia were thus just as informative for optimising the design‐build‐test‐learn (DBTL) cycles as the engineering successes (Erpf et al. 2025; James et al. 2025; Zhao et al. 2023a).

Amongst the independent synthetic chromosome constructs, parallel advances in quality control and repair further strengthened the Sc2.0 framework. For example, pooled PCRtag Mapping (PoPM) (Wu et al. 2017) enabled high‐throughput validation of synthetic DNA segments that are integrated into the native genome, while CRISPR‐based debugging platforms, such as the CRISPR Directed Biallelic URA3‐assisted Genome Scan (CRISPR d‐BUGS) protocol, facilitated rapid correction of synthetic chromosome design defects (Zhao et al. 2023a).

The CRISPR d‐BUGS system works by exploiting directed mitotic recombination in heterozygous diploid yeast (carrying one synthetic and one wild‐type chromosome). By using CRISPR‐Cas9 to induce double‐strand breaks on the synthetic chromosome, the cell is forced to become homozygous for either the synthetic or wild‐type sequence in specific regions. By screening these ‘recombinant’ clones for the recovery of wild‐type growth, it is then possible to precisely map a defect, using watermarked PCRtags designed into the synthetic DNA, to a small genomic window, therefore narrowing a ‘bug’ down to a few hundred base pairs (Zhao et al. 2023a). This has been showcased following the construction of an *S. cerevisiae* strain bearing synII, where d‐BUGS traced the cause of a fitness defect to two loxPsym sites located in the 3′ UTR of the *SHM1* gene that encodes for an essential enzyme responsible for carbon metabolism (Zhao et al. 2023a). In another example, the CRISPR d‐BUGS method was employed to resolve fitness defects in an *S. cerevisiae* strain bearing synXVI (Goold et al. 2025). The synthetic chromosome defect was also identified as loxPsym sites that inadvertently disrupted the 5′ UTRs of the essential genes *CTR1* and *GIP3*, encoding for proteins that are necessary for copper transport and various cellular processes, respectively (Goold et al. 2025).

These engineering troubleshooting tools constitute a scalable genome debugging infrastructure that will now also support ongoing consolidation efforts to combine all synthetic chromosomes into a single host cell. Furthermore, the versatile suite of genome engineering methodologies that emerged from Sc2.0 has laid the operational foundation towards a consolidated synthetic eukaryotic genome and the development of future synthetic yeast strains.

### 4.2 Towards a Consolidated Sc2.0 and a Highly Refactored Sc3.0

The consolidation of all sixteen synthetic chromosomes into a single cell represents the next grand challenge. To date, six full synthetic chromosomes and one synthetic chromosome arm have been combined, representing approximately one‐third of the haploid *S. cerevisiae* genome (Zhao et al. 2023a). As anticipated, both recessive and dominant designer‐induced fitness defects were characterised in the semi‐synthetic genome, whereby use of CRISPR d‐BUGS enabled systematic mapping and resolution of these identified defects (Zhao et al. 2023a). The conceptual and practical frameworks for full consolidation of Sc2.0 chromosomes are thus emerging. A review by Schindler et al. (2024) further summarises the goals to reach consolidated synthetic genomes, with concepts of yeast hierarchical mating and chromoduction that provide feasible routes toward a fully synthetic Sc2.0 genome and beyond.

Whereas Sc2.0 is still structured to preserve the native gene content with relatively minimal genome rearrangement, the development of Sc3.0 is further envisioned to embrace even more radical genome refactoring rather than simply mirroring native gene order (Dai et al. 2020; Erpf et al. 2025; Schindler. 2020). What is promising is that large‐scale refactoring of the native *S. cerevisiae* genome, although not synthetic versions, has already been proven to have minimal effects on strain fitness, even when all 16 native chromosomes are either fused or fractioned (Luo et al. 2018; Shao et al. 2018; Ueda et al. 2012). To ensure the industrial viability of synthetic yeast cell lines, it is essential to rigorously evaluate cellular fitness under both basal and challenging environmental conditions. The next generation of synthetic genome platforms should thus be inherently customisable to preserve such phenotypic plasticity across varying environmental/industrial culturing conditions.

The capacity to reconfigure the *S. cerevisiae* genome as a modular, designer substrate underscores the potential to produce industrially relevant synthetic yeast strains. In this context, neochromosomes emerge as a potentially valuable platform for future innovations, with existing design principles already demonstrating the stable maintenance of such genomic structures. Such modular and orthogonal chromosome platforms can both (i) facilitate refactoring of yeast synthetic genomes, and/or (ii) enable alternative engineering routes for introducing entirely new genetic functionalities via a dedicated orthogonal landing pad. Therefore, neochromosomes prove to serve not merely as auxiliary elements for future genome assembly but also expand the application of *S. cerevisiae* to create novel, build‐to‐spec functionalities.

## 5 Build‐to‐Spec Neochromosomes

Although the concept of yeast as genome factories has been demonstrated for decades, the de novo construction of supernumerary designer chromosomes to harbour such a large cargo of heterologous gene sets is still only in its infancy. Neochromosomes, therefore, represent engineered extra‐chromosomal elements that can be introduced independently of the native genome and subsequently converted into true linear chromosomes. Given advances in synthetic genome design, neochromosomes represent a relatively blank canvas and a new frontier to substantially expand the metabolic and functional capacity of engineered yeast strains.

The development of a neochromosome chassis as an orthogonal genomic sandbox is guided by a few key design requirements to replicate a natural eukaryotic chromosome. Similar to yeast shuttle vectors and YACs, core elements include a yeast centromere and ARSs to support timely chromosomal segregation and DNA replication, respectively. In addition, a telomere seed sequence (i.e., telomerator) (Mitchell and Boeke 2014) serves as an ideal synthetic element to induce linearisation of the neochromosome and subsequently promote the synthesis of functional telomeres in vivo (Schindler et al. 2023). Moreover, the size of the synthetic chromosome and distribution of replication origins (ARSs) should also be considered. While chromosomes below 100 kb are suggested to become unstable in yeast models, increasing the size to approximately 150 kb, at least in the case of circular chromosomes, results in mitotic stability comparable to smaller endogenous (linear) chromosomes in the native genome (Murray et al. 1986). To avoid replication delays, ARS sequences are distributed on average between ~30–40 kb across the native *S. cerevisiae* genome (Dhar et al. 2012; Postma et al. 2021). However, it has been demonstrated that successful replication can be achieved in synthetic chromosome constructs when ARSs are spaced ~100–200 kb apart (Noskov et al. 2012). These general design principles and considerations, together with the expanding suite of molecular tools that enhance neochromosome modularity and tractability, provide a practical framework for engineering heavily tailored synthetic yeast strains. The following section examines how such neochromosome‐based approaches will expand the design space for future synthetic genome innovation.

### 5.1 Pan‐Genome and Metagenomic Neochromosomes

Most foundational research on *S. cerevisiae* has focused almost exclusively on the laboratory lineage S288c and its affiliated strains. However, this obscures the vast phenotypic diversity and fitness found in hundreds of lineages adapted to distinct ecological and industrial niches. Much of this phenotypic diversity arises from intraspecific genetic variation and accessory gene clusters absent from defined lineages, meaning that reliance on S288c alone obscures the broader functional repertoire of the species. To address this gap, the concept of pan‐genome neochromosomes (PGNCs) offers a compelling strategy to integrate accessory genetic diversity into a tractable host cell.

This was demonstrated as a sub‐component of Sc2.0 through the construction of a 211 kb PGNC containing 75 open reading frames (ORFs) from diverse *S. cerevisiae* strains that were identified to be absent in the engineered test strain (Kutyna et al. 2022). Notably, the PGNC was fully compatible with the Sc2.0 design framework, including SCRaMBLE‐mediated rearrangement that enabled direct exploration of genotype‐phenotype relationships within the accessory genome. The stable expression of this neochromosome increased phenotypic flexibility in the engineered *S. cerevisiae* strains, including expanded carbon source utilisation and enhanced stress tolerance, while also revealing key design principles governing neochromosome stability, replication origin placement, and trait mapping.

**Figure 2:**
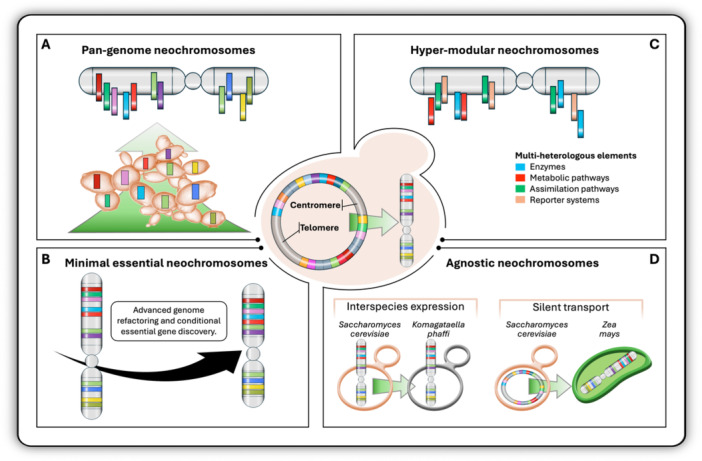
Conceptualising the utility of purpose‐built neochromosomes across fundamental eukaryotic biology discovery and bioindustrial applications. (A) Pan‐genome neochromosomes enable the absorption of lineage‐wide accessory gene clusters absent from the recipient engineered host yeast strain. This expands the genomic landscape of the engineered strain, diversifying or enhancing its phenotype under various culturing conditions. (B) Minimal essential neochromosomes provide a defined genomic landing pad for large‐scale genome refactoring. Such platforms allow the rational reduction of genetic redundancy while consolidating conditionally essential genes required to maintain metabolic fitness across variable environmental or industrial culturing conditions. (C) Hyper‐modular neochromosome chassis establish a dedicated chromosomal platform in which the integration of multi‐heterologous genetic elements can expand the metabolic landscape of the engineered yeast strain without altering the structural integrity and regulatory balance of the native genome. (D) Agnostic neochromosomes extend *Saccharomyces cerevisiae* further as a versatile in vivo genome foundry. These platforms may (i) support modular, orthogonal gene expression systems governed by conserved or synthetic transcriptional regulators that function across diverse host species, or (ii) serve as assembly scaffolds for heterologous gene cassettes and essential chromosomal elements that can be transferred as linear, functional chromosomes into a target organism (silent transport).

Following the initial 211 kb PGNC (Kutyna et al. 2022), a 1.024 Mb synthetic accessory chromosome (synAC) was reported, which encoded 183 non‐homologous genes and 359 orthologous genes from the broader *S. cerevisiae* pan‐genome (Ma et al. 2024). This synAC offered complex traits, including glutarate utilisation, improved low‐temperature tolerance, as well as the discovery of new biosynthetic products that are not prevalent in the reference engineered strain (Ma et al. 2024). With an ever‐expanding *S. cerevisiae* pan‐genome (Loegler et al. 2024; Peter et al. 2018; Wang et al. 2024), such platforms serve as a well‐characterised resource to support the design of supernumerary neochromosomes and improved bio‐industrial yeast strains that will capture all the most desirable elements amongst *S. cerevisiae* lineages (Figure 2A).

Looking beyond *S. cerevisiae* lineages alone, synthetic metagenomic neochromosomes offer an evolved concept. By capturing essential functions from diverse microbial consortia, we can introduce novel functionality to perform a desired biotechnological task (Belda et al. 2021), along with the potential for highly expanded biological functions in the engineered yeast strain (Gong et al. 2022; Morales and Dujon 2012). Similarly, engineered inter‐species yeast hybrids demonstrate that traits can be consolidated into one host, such as cold tolerance and faster xylose utilisation through allopolyploid formation (Peris et al. 2020). The concept of such a synthetic metagenome that is captured on neochromosomes could shed light on interspecies interactions within a defined environmental niche, or having a single strain that combines all the essential biological features to perform a dedicated task (Belda et al. 2021). Neochromosomes ultimately offer a more modular and controllable alternative for diverse biotechnological applications. Moreover, with the aid of a yeast host having a highly refactored or rearranged synthetic genome (i.e., Sc3.0), the introduction of pan‐genomic and metagenomic neochromosomes will enable the functional contributions of entire microbial communities to be encoded within a single eukaryotic genome.

### 5.2 Towards Minimal Essential Neochromosomes

The realisation of fully synthetic yeast genomes in Sc2.0 and for Sc3.0 requires systematic rationalisation and a deeper understanding of genome‐scale biological complexity. Cellular function originates from multiple layers of interconnections among genetic elements, regulatory cycles, and metabolic pathways encoded by the native yeast genome. The concept of minimal synthetic genomes thus offers a new testbed to explore and rationalise this complexity at the systems level (Xu et al. 2023).

A relevant example of this concept emerged from an engineering component within Sc2.0, which included the genome‐wide relocation of tRNA genes onto a ~190 kb neochromosome. This further facilitated systematic removal of introns to reduce sequence complexity, uncovering context‐dependent regulatory roles of tRNA genome architecture extending beyond their canonical functions (Schindler et al. 2023). Moreover, the tRNA neochromosome utilised Dre‐rox recombination sites to provide an orthogonal system to conventional LoxPsym recombination sites for SCRaMbLE. Altogether, this work showcased a method for large‐scale refactoring of the native genome and demonstrated that highly dispersed genomic elements can be modularised into an independent genomic unit. Developing minimal genomes is particularly relevant given the need to remove genetic redundancy whilst preserving conditionally essential genes required for defined environmental or stress conditions (Figure 2B). Strains harbouring reduced, essential genomes are hypothesised to exhibit improved fitness due to reduced transcriptional and translational burdens (Xu et al. 2023). However, a careful balance should be achieved as excessive reduction can reduce fitness across diverse culturing environments, which is particularly critical if minimal or refactored genomes are to support applied biotechnological innovation.

The removal of large‐scale genomic redundancy and the compartmentalisation of essential functions on dedicated chromosomal platforms would enable iterative metabolic rewiring or pathway swapping (Boonekamp et al. 2022; Yu et al. 2022). Such approaches provide opportunities to improve yeast metabolism, explore evolutionary constraints, and interrogate complex metabolic pathways from higher eukaryotes within a simplified and tractable eukaryotic model. These interventions would allow the representation of neochromosomes as a foundational engineering platform for deconstructing, reorganising, and rebuilding yeast genomes.

### 5.3 Hyper‐Modular Neochromosomes for Tailored Heterologous Expression

Although *S. cerevisiae* has long served as a well‐established microbial cell factory to produce biofuels, pharmaceuticals, fine chemicals, and other high‐value products, extensive integration of heterologous genetic material into its native genome can compromise cellular fitness and genomic integrity. In contrast, modular plug‐and‐play neochromosome scaffolds offer an orthogonal genomic platform for the multiplexed introduction of heterologous genes, pathways, and regulatory systems while preserving the structural and regulatory integrity of the host native genome (Figure 2C). This strategy decouples metabolic innovation from intrinsic evolutionary and regulatory constraints, thereby enabling the installation of expanded or entirely novel biological capabilities.

Such a modular, de novo‐designed neochromosome chassis concept has been experimentally validated. The study by Postma et al. (2021) demonstrated the synthesis of modular, yet largely non‐coding neochromosome scaffolds of 50 and 100 kb in *S. cerevisiae*. Upon stable integration of the neochromosome chassis in the host cell, further integration of a 35 kb glycolytic cassette comprising 13 transcriptional units yielded 85 kb and 135 kb neochromosome structures, which were shown to be stably maintained and resulted in enhanced phenotypic flexibility of the engineered *S. cerevisiae* strain (Postma et al. 2021). This demonstrated the ability to construct these new‐to‐nature synthetic chromosomes in vivo using yeast HR machinery, establishing a scalable route for tailored neochromosome structures that will ensure their stable integration as an annexed genome component in the engineered yeast strain.

The ability to install large, multi‐gene modules on a dedicated chromosomal platform has far‐reaching implications for yeast biotechnology. Neochromosomes offer the potential to accommodate complex biosynthetic pathways that can enable various applications such as enhanced production of high‐value compounds, redirection of metabolic fluxes for tailored fermentation outcomes, deployment of biosensor arrays for environmental pollutant detection, or improved tolerance to diverse industrial cultivation conditions. For example, the incorporation of exogenous biological functions supporting the complete saccharification of complex carbohydrate substrates, together with the required assimilation pathways for efficient utilisation of reducing sugars, offers a promising framework for consolidated bioprocessing (CBP) across an expanded substrate spectrum in biorefinery settings (Jacob et al. 2022; Liu et al. 2016; Mert et al. 2016; Oh & Jin 2020).

### 5.4 Agnostic Neochromosomes for Cross‐Species Functionality

Potentially the most ambitious vision includes synthetic chromosome scaffolds that are engineered to function across species barriers, namely agnostic neochromosomes. This lies in the development of truly orthogonal genomic chassis for high‐performance bioengineering across non‐conventional yeasts, and eventually, expanding to higher eukaryotic cell types. These constructs can be designed to operate independently of a single host species by incorporating conserved chromosomal elements and modular regulatory architectures. Such features enhance functional predictability, support portability across diverse eukaryotic systems, and enable deployment in multiple organisms. This assembly‐and‐transfer concept builds on earlier milestones in synthetic biology, where *S. cerevisiae* served as the in vivo genome foundry for a range of heterologous gene clusters and genomes from other donor organisms (Brown et al. 2017; Camellato et al. 2024; Liu et al. 2025; Mitchell et al. 2021; Pinglay et al. 2022; Zhao et al. 2023b) (see further examples in Table 1). *Saccharomyces cerevisiae* can now be considered a universal living genome foundry where neochromosomes can be constructed and transferred into other eukaryotic hosts.

Two complementary design strategies can be envisioned for agnostic neochromosomes. In one model, chromosomes are built from multi‐modular, orthogonal gene clusters regulated by conserved or synthetic transcriptional systems (Zuo et al. 2025), enabling partial or full functionality across multiple hosts (Figure 2D). In a second model, neochromosomes act as transport vectors carrying gene cassettes and essential chromosomal elements that remain transcriptionally inactive in the assembly host but become functional only on transfer to a target cell (Figure 2D). The latter strategy has the advantage of reducing host cell burden during construction while preserving portability and functional specificity.

Despite these visions, some challenges do remain. Species‐specific differences in chromatin organisation, their epigenetic regulation, transcriptional machinery, and pathway connectivity may compromise the stability and function of the neochromosome platform. Addressing these constraints will require improved insulation strategies and a deeper understanding of conserved regulatory principles between species. Moving beyond the foundational successes in *S. cerevisiae* requires overcoming mechanical barriers and the limited availability of characterised synthetic chromosome ‘hardware’ in other organisms (CENs, ARSs, telomere seed sequences, etc.). As an example, unlike the short, conserved ~125 bp point centromeres of *S. cerevisiae*, the centromeres in yeasts such as *K. phaffi* are larger and regional, spanning over several kilobases. Moreover, a centralised database comparable to the *Saccharomyces* Genome Database (SGD) or the Yeast Toolkit (YTK) (Lee et al. 2015) is not readily available for other yeast species. However, modular engineering toolkits for non‐conventional yeasts are expanding, such as for *Y. lipolytica* (Jiang et al. 2024; Larroude et al. 2019), *K. phaffii* (Claes et al. 2024; Obst et al. 2017), and *Kluyveromyces marxianus* (Rajkumar et al. 2019), all of which prove to be useful resources to serve as homologs of *S. cerevisiae* neochromosome parts. Progress in this area will be strongly enabled by integrated genomics and systems biology frameworks across eukaryotes and further accelerated through the convergence of synthetic biology with artificial intelligence (AI) to drive frontier discoveries. These platforms could help to redefine the portability of biological function in the age of synthetic genomics.

It is positive to note that initial progress in non‐conventional yeast neochromosomes is already evident. The work by Abramczyk and colleagues designed a 15–25 kb ‘nano‐chromosome’ for the yeast *K. phaffi* (Abramczyk et al. 2023). While the nano‐chromosome is stable over many generations, its persistence requires a ΔKU70 background to compromise the hosts NHEJ pathway, which otherwise leads to integration or instability. Therefore, expanding the concept of ‘silent transport’ of agnostic neochromosomes (Figure 2D) represents a valuable strategy to address this challenge. In this framework, *S. cerevisiae* serves as a ‘genome foundry’ to assemble the construct *via* its efficient HR machinery before the complete, functional neochromosome is transferred to the target host. Notably, such an approach will require an integration of dual‐species chromosomal ‘hardware’ in the neochromosome. Alternatively, recent research into reprogramming *S. cerevisiae* to recognise humanised telomeric repeats (D'Angiolo et al. (2026) opens new possibilities for developing synthetic elements that facilitate interspecies expression and stability.

### 5.5 Considerations to Ensure Host Cell Fitness and Neochromosome Stability

Beyond fundamental parameters such as chromosome size and ARS spacing mentioned before, the broader impact of neochromosome integration on cellular fitness remains a critical consideration for synthetic genomics. While the creation of expansive ‘genomic real estate’ using neochromosomes offers a promising route for introducing heterologous pathways or amplifying native gene copies, such kilobase‐to‐megabase scale additions may impose significant fitness burdens. This is particularly evident when synthetic genes are expressed constitutively rather than according to cellular demand. Such intensive expression can sequester various cellular resources, creating ‘sinks’ in ATP and precursor metabolites while potentially overloading the hosts protein‐folding and translational machinery (Kastberg et al. 2022; Lin et al. 2023). Such an intensive resource competition triggers global stress responses and forces a reallocation of cellular energy, leading to reduced growth rates, decreased genetic stability, and lower overall biosynthetic productivity (Kastberg et al. 2022).

Furthermore, large‐scale genomic additions can disrupt nuclear spatial organisation or induce aneuploidy‐related stress, such as in the case of the tRNA neochromosome, where a transcriptional burden prompted spontaneous ploidy doubling to buffer the resulting physiological imbalances (Schindler et al. 2023). Even the addition of large non‐coding regions can induce fitness defects, as showcased by Postma et al. (2021), where the addition of a ~50 to ~100 kb neochromosome in *S. cerevisiae* led to decreased growth rates compared to the wild type (Postma et al. 2021). While spatial organisation will be an ongoing challenge to resolve, some level of resolution for other avenues of metabolic burdens imposed by large genomic additions includes the implementation of integrated regulatory circuits, inducible control systems, or refined response elements targeting post‐translational modifications and metabolic feedback mechanisms (Lin et al. 2023; Ni et al. 2023). However, the specific strategies required to mitigate these metabolic burdens must likely be assessed on a case‐by‐case basis.

Structural modularity of neochromosomes should also be acknowledged to introduce risks of impaired host cell fitness. For example, the misplacement of genetic markers or loxPsym sites near 5′ UTRs or 3′ UTRs of essential genes has been shown to disrupt native gene expression or cause unintended read‐through (Erpf et al. 2025; Goold et al. 2025; Zhao et al. 2023a). For this reason, while SCRaMbLE‐associated sites facilitate modularity, limiting their density may be necessary to ensure the fitness of the engineered strain (Erpf et al. 2025). Finally, design considerations to facilitate segregation of artificial chromosomes are of importance to ensure their stable maintenance in a host cell. It has been shown, for example, that incorporating at least 500 bp of native sequences that are proximal to the CDEIII centromeric elements can significantly improve chromosome stability, as shown in the case of synthesising the synVIII chromosome in *S. cerevisiae* (Lauer et al. 2023). Also, the development of synthetic kinetochores holds promise for improving the segregation dynamics of synthetic genomic constructs (Lacefield et al. 2009; Postma et al. 2021), which warrants the inclusion of such genomic design considerations to ensure the mitotic stability of synthetic chromosomes and neochromosomes.

## 6 The Enduring Legacy of Synthetic Yeast Genomics

Over the decades, *S. cerevisiae* exemplifies how a single eukaryotic organism can redefine the boundaries of genome engineering and biological design: First as a fermentative agent, then as a foundational eukaryotic model, and ultimately as a programmable genome foundry. What began as incremental, gene‐centric modification of *S. cerevisiae* on its native genome (Sc1.0) has evolved into the deliberate writing, restructuring, and repurposing of entire eukaryotic genomes (Schindler 2020). Viewed through the lens of its long‐term impact, the Sc2.0 project is best understood as the enabler that made genome‐scale interrogation of eukaryotic biology experimentally tractable. This scientific maturation from merely manipulating genes within a fixed genome to treating the genome as an engineered system represents one of the most significant conceptual advances in modern yeast biology.

The suite of DBTL frameworks and associated engineering toolkits now at the disposal of the modern synthetic biologist allows for the yeast genome to be treated as a programmable substrate. This progression naturally extends beyond whole‐genome synthesis toward advanced genome design. In the context of a Sc3.0, it is not defined by a single canonical synthetic genome, but by a design philosophy in which genomes are manipulated and tailored to explore fundamental constraints and opportunities in eukaryotic biology (Dai et al. 2020). At the same time, unresolved questions about the stability and the necessary plasticity that must be retained in a synthetic genome for the strain to be utilised under industrial conditions underscore the need for continued innovation in chromosome design principles. This legacy aligns closely with emerging perspectives that position synthetic genomes as bioinformational substrates. Such structures would not only encode biological function but also generate, transmit, and transform information through their interaction with cellular and environmental contexts (Pretorius et al. 2025).

**Figure 3:**
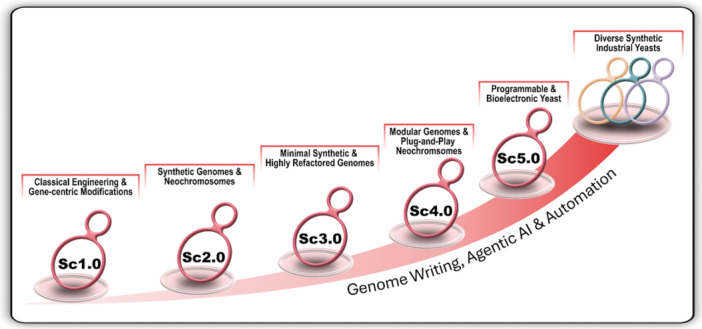
The ascent of synthetic yeast biology from its origins as a classical genetic engineering platform (*Sc1.0*) to sophisticated genome‐scale design frameworks. The synthetic yeast genome project (*Sc2.0*) laid the foundation for rational genome redesign, delivering advanced engineering toolkits, systematic debugging strategies, and key conceptual and technical advances in neochromosome construction that define the current state of synthetic genome biology. Building on these achievements, the field progresses toward *Sc3.0*, characterised by a highly refined and controllable *Saccharomyces cerevisiae* genome layout achieved through the integration of automation, AI, and programmable platforms. This evolution extends into *Sc4.0*, which focuses on consolidating these synthetic designs to create specialised yeast genome chassis optimised for high‐performance metabolic flux and the manufacturing of value‐added products. In parallel, *Sc5.0* envisions the hybridisation of yeast synthetic biology with electronic systems to develop programmable bioelectronic platforms for sensing, computation, and actuation. Ultimately, these benchmarked foundations in *S. cerevisiae* facilitate the expansion of synthetic genomics into non‐conventional species, enabling the development of robust, customisable, and species‐agnostic genome chassis across a diverse range of industrially relevant yeasts.

One can envision a rapid acceleration in the engineering of synthetic genomes for industrially vital non‐conventional yeasts in the coming decade. Would it not be a spectacular achievement to report on Sc3.0 and synthetic genomes for non‐conventional, industrially relevant yeast strains on the 50th anniversary of the Journal Yeast? Such a vision extends synthetic genome engineering beyond a single model species toward a diversified ecosystem of industry‐ready and purpose‐built yeast chassis (Erpf et al. 2025) (Figure 3). Fully synthetic genomes of various yeast cell factories, each possessing distinct metabolic capabilities, secretion profiles, and environmental tolerances, would collectively expand the design space available to biotechnology. In this phase, genome writing becomes a means to encode function at scale, enabling industries to deploy customised synthetic genomes that are optimised for highly defined substrates, products, and operating conditions. Core genomes may provide standardised, evolutionarily stable cellular infrastructure, while neochromosomes encode novel biosynthetic pathways, conditional essential genes, or multiplexed heterologous gene cassettes. Such platforms promise faster strain development, reduced redevelopment costs, and greater interoperability across sectors ranging from biomanufacturing to environmental monitoring.

Realising these ambitions will depend on the convergence of multidisciplinary scientific and engineering platforms, including synthetic genomics, agentic AI, and advanced automation. Agentic AI systems, capable of goal definition and iterative learning, offer a natural extension to DBTL pipelines on the genome scale (Bandi et al. 2025; Wang et al. 2025b). By integrating multi‐omics data streams with phenotypic and process‐level feedback, AI agents can infer latent design rules, propose chromosome‐level redesigns, and learn from both successful and failed experiments. In doing so, they promise to transform synthetic genome engineering from an expertise‐intensive discipline into a scalable and partially autonomous engineering practice. As a result, AI‐guided genome rearrangements, along with phenotypic and sequencing validation facilitated by advanced biofoundry automation, will transform strain optimisation into a continuous, data‐driven process scalable for industrial applications (i.e., Sc4.0).

The convergence of multidisciplinary science, engineering, and techno‐economic interventions aligns with broader trends toward the integration of bioinformatics and biosynthetic yeast strains, in which biological systems are engineered as programmable platforms for sensing, computation, and actuation (namely bioelectronic yeasts; Sc5.0; Figure 3) (Erpf et al. submitted; Pretorius et al. 2026).

In this emerging landscape, yeast stands as both a testbed and a prototype; a eukaryotic system in which the principles of synthetic genome design can be developed and ultimately translated into even broader biological and technological infrastructures. The coming decades will determine not simply how well we can write genomes, but how effectively genome‐scale design is integrated into the wider information and engineering ecosystems that define modern biotechnology and scientific imagination.

## Conflicts of Interest

The authors declare no conflicts of interest.

## Data Availability

Data sharing is not applicable to this article as no datasets were generated or analysed during the current study.
